# Targeting the cGAS-STING Pathway: Modulating Inflammation, Oxidative Stress, and Autophagy for Novel Depression Therapies

**DOI:** 10.2174/011570159X386374250623095520

**Published:** 2025-07-11

**Authors:** Wen Ma, Shanshan Chu, Yumei Ma, Sutian Wang, Xuehai Ma

**Affiliations:** 1 Xinjiang Normal University, Urumqi, China;; 2 The Friendship Hospital of Ili Kazakh Autonomous Prefecture, Ili, China;; 3 State Key Laboratory of Swine and Poultry Breeding Industry, Guangdong Key Laboratory of Animal Breeding and Nutrition, Institute of Animal Science, Guangdong Academy of Agricultural Sciences, Guangzhou, China;; 4 Xinjiang Key Laboratory of Mental Development and Learning Science, College of Psychology, Xinjiang Normal University, Urumqi, China

**Keywords:** cGAS-STING, depression, neuroimmunity, inflammation, oxidative stress, autophagy, therapeutic strategy, physical exercise

## Abstract

The pathological mechanisms underlying depression, a prevalent mental disorder, remain only partially elucidated despite extensive research efforts. Recent advancements have underscored the pivotal roles of multiple biological processes in the onset and progression of depression, including inflammation, oxidative stress, and autophagy. Inflammation is associated with the disruption of neurotransmitter systems and neural plasticity, whereas oxidative stress contributes to neuronal damage and impaired brain function. Moreover, moderate autophagy is essential for maintaining neuronal health. Dysregulation of autophagy may lead to the accumulation of damaged proteins and organelles, which can exacerbate depressive symptoms. Among the various molecular pathways involved, the cGAS-STING signalling pathway has emerged as a key regulator of these processes. Traditionally known for its role in detecting cytosolic DNA and initiating innate immune defences against pathogens, the cGAS-STING pathway has recently been implicated in regulating inflammatory responses, oxidative stress levels, and autophagy in the central nervous system. This dual function positions the cGAS-STING pathway as a potential link between immune dysregulation and the neurobiological foundations of depression. This paper offers a systematic overview of existing studies on the role of the cGAS-STING pathway in inflammation, oxidative stress, and autophagy within the central nervous system, particularly in the context of depression. The review reveals how modulation of the cGAS-STING pathway may influence these critical biological processes and thereby ameliorate depressive symptoms. Furthermore, the review discusses the therapeutic potential of targeting the cGAS-STING pathway and offers promising research directions. Ultimately, this paper aims to provide novel insights and approaches for developing more effective treatments for depression.

## 1 INTRODUCTION

Depression is a psychiatric disorder characterized by persistent low mood, anhedonia, cognitive deficits, sleep disturbances, appetite loss, and suicidal ideation, all of which significantly impair individuals' psychosocial functioning and quality of life. In response to increasing societal and occupational stressors, the prevalence of mental illness has surged, with estimates indicating that over 350 million individuals globally (approximately 4.4% of the population) are affected by depression [1, 2]. Moreover, major depressive disorder (MDD) is closely associated with obesity, cardiovascular disease, and premature mortality. The World Health Organization (WHO) has identified depression as the third leading contributor to the global disease burden. Additionally, the COVID-19 pandemic has further increased the incidence of depression by more than 25% worldwide [3]. Over the past decade, the rate of depression has risen by approximately 18%, and it is projected that by 2030, depression will become the leading cause of disability. Contemporary biomedical models conceptualize depression as a dysregulation of neural networks involving alterations across widely distributed brain regions [4]. Antidepressants are the primary treatments for moderate to severe depression. Unfortunately, traditional antidepressants are not only slow-acting and associated with multiple side effects, but more than one-third of patients do not respond to these medications, suggesting that the pathogenesis of depression may involve additional factors [5]. Therefore, there is an urgent need to explore the pathophysiological mechanisms of depression and identify novel therapeutic targets.

Inflammatory processes are linked to the pathophysiology of depression [6]. Strong evidence indicates elevated concentrations of inflammatory cytokines, such as interleukin 6 (IL-6), tumor necrosis factor α (TNF-α), and C-reactive protein (CRP), are consistently higher in individuals with depression compared to control subjects [7]. The increased levels of inflammation have been observed both peripherally and centrally in individuals with depression. Mechanistically, early life stress and environmental stressors upregulate pro-inflammatory cytokine levels, which subsequently interact with secondary mediators, activate microglia, and influence glutamatergic and other signalling pathways. Numerous trials have evaluated the efficacy of anti-inflammatory agents in the treatment of depression [2]. Inflammation is closely linked to various pathophysiological processes, including oxidative stress, mitochondrial dysfunction, and autophagy. These processes have also been implicated in the onset and progression of depression. Under pathological conditions, inflammation and oxidative stress reinforce each other, creating a coactivation state [8]. Markers of oxidative stress, such as 8-OH 2-deoxyguanosine and F2-isoprostanes, are significantly elevated in MDD [9]. Notably, excessive reactive oxygen species (ROS) that contribute to oxidative stress primarily originate from mitochondria. Elevated levels of mitochondrial ROS have been observed in depression, suggesting mitochondrial dysfunction [10]. Furthermore, ROS can also trigger autophagy, an evolutionarily conserved process involved in degrading surplus proteins and organelles while maintaining homeostasis [11]. Autophagy dysfunction has been observed in patients with MDD and animal models of depression [12].

The cGAS-STING pathway has been shown to regulate inflammation, oxidative stress, and autophagy processes [13]. In original studies, researchers found that microbial DNA induces interferon production by activating the cGAS-STING signalling axis [14]. Since then, a series of studies have demonstrated that cGAS-STING signalling is involved in the induction of inflammation, regulation of redox homeostasis, mitochondrial dysfunction, and programmed cell death (including autophagy and pyroptosis) [13]. Moreover, dysregulation of the cGAS-STING pathway contributes to the development of various mental disorders and neurodegenerative diseases [15]. Recent clinical and preclinical evidence indicates that the cGAS-STING signalling axis is involved in depressive symptoms [16, 17]. Several studies suggest a promising role for cGAS-STING signalling in the treatment of depression [18, 19]. In addition, many studies have found exercise to be one of the most effective treatments for depression. Recently, several studies have shown that exercise is involved in the regulation of cGAS-STING activity, thus further affecting inflammation and tissue damage [20, 21]. Consequently, targeting cGAS-STING may be a promising approach for both the treatment and prevention of depression. This review aims to elucidate the various roles of cGAS-STING signalling in depression, specifically the potential mechanisms through which it modulates associated pathological processes implicated in the pathogenesis of depression, while also offering novel insights for developing treatment strategies.

An electronic search of SCOPUS, PubMed, and Web of Sciences databases was performed to recognize the relevant research available until 15 January 2025 in order to examine the cGAS-STING findings in depression. Search terms were “STING” or “cGAS-STING” or “Stimulator of interferon genes” or “TMEM173”, “Inflammation” or “ innate immune” or “Oxidative Stress” or “Autophagy”, and “depression” or “central nervous system” or “neurodegenerative disease” or “Brain damage”. The search was further extended by a snowball search and hand searching by using Google Scholar.

The following inclusion criterion was adopted: (1) studies published in English; (2) papers reporting cGAS-STING signalling in depression, central nervous system, neurodegenerative disease, or brain damage with a description of the findings; (3) original articles that report the correlation between cGAS-STING signalling and inflammation, innate immune response, oxidative stress, or autophagy. The following exclusion criteria were applied: (1) scientific articles not published in the English language; (2) full text not available. For duplicate studies, only the article with more detailed information was included.

## 2 OVERVIEW OF cGAS-STING SIGNALLING PATHWAYS

### 2.1 The basis of cGAS-STING Signalling

Cyclic GMP-AMP synthase (cGAS) is a recently discovered pattern recognition receptor (PRR) that detects viral, endogenous, mitochondrial, and genomic DNA within the cytoplasm, playing a crucial role in innate immunity. Chen *et al.* were the first to identify cGAS and elucidate how DNA stimulates immune and inflammatory responses. Upon DNA binding, cGAS undergoes conformational changes that facilitate the synthesis of cyclic GMP-AMP (cGAMP). This secondary messenger is subsequently recognized by the stimulator of interferon genes (STING), which is located in the endoplasmic reticulum [22]. STING, characterized by Ishikawa and Barber, is an ER-associated protein that mediates interferon induction in response to viral and intracellular DNA stimulation [23]. Activated STING translocates to the Golgi apparatus, where it recruits TANK-binding kinase 1 (TBK1) and interferon regulatory factor 3 (IRF3) to form a signalling complex [24]. TBK1 phosphorylates and promotes the oligomerization of IRF3. Consequently, phosphorylated IRF3 migrates into the nucleus, initiating the transcription of type I interferons (IFNs) and interferon-stimulated genes (ISGs) that exert antiviral effects. Initially, researchers believed that IFN induction was the primary function of cGAS-STING signalling. However, Gui and colleagues demonstrated that the induction of autophagy is an ancient and highly conserved function of the cGAS-STING pathway, which evolutionarily predates the emergence of the IFN pathway in invertebrates [25]. Upon binding to cGAMP, STING undergoes a conformational change and translocates from the ER to the Golgi apparatus *via* the ER-Golgi intermediate compartment (ERGIC). Within the ERGIC, STING initiates autophagy. The translocation process of STING relies on the COP-II complex and ARF GTPases. The ERGIC-containing STING serves as a membrane source for LC3 lipidation, promoting the formation of autophagosomes. Ultimately, autophagosomes fuse with lysosomes, leading to the degradation of their contents. Gui *et al.* demonstrated that the LC-3-interacting regions (LIRs) of STING directly interact with LC3, inducing ATG5-dependent non-canonical autophagy.

Furthermore, evidence suggests that invertebrate STING triggers autophagy, but not IFN, in response to cGAMP stimulation, indicating that autophagy induction may be a fundamental function of the cGAS-STING signalling pathway [25]. Ultimately, STING located within autophagosomes and STING derived from the Golgi apparatus both move to the lysosome, where STING degradation occurs. In the absence of cGAMP stimulation, retrograde transport mediated by COPI vesicles impairs the steady-state translocation of STING through the secretory pathway, as retrograde transport is enhanced by the interaction between STING and SURF4 [26].

### 2.2 Physiological Functions of the cGAS-STING Pathway

The cGAS functions as a versatile sensor for double-stranded DNA (dsDNA), enabling the innate immune system to detect a broad spectrum of pathogenic microorganisms. This role has been validated through genetic studies using cGAS-deficient mice, which exhibit impaired IFN responses to infections by DNA viruses such as herpes simplex virus, adenovirus, and vaccinia virus [27]. These mice show elevated viral loads and increased mortality when exposed to HSV-1, vaccinia virus, or murine gammaherpesvirus, underscoring the critical importance of the cGAS-STING pathway in antiviral defence. Interestingly, cGAS- and STING-deficient mice also display heightened susceptibility to RNA virus infections despite normal IFN production *in vitro* [28]. This observation suggests that the cGAS pathway may play a role in maintaining basal interferon levels essential for controlling both DNA and RNA viruses *in vivo*. Alternatively, cellular damage induced by RNA viruses could lead to the release of endogenous DNA, subsequently activating cGAS and triggering an immune response.

In addition to DNA viruses, cGAS is crucial for sensing retroviruses, including HIV-1 and HIV-2 [29]. Upon entry into macrophages and dendritic cells, reverse transcription converts viral RNA into DNA, which integrates into the host genome. Typically, retroviruses evade strong innate responses; however, compromised capsid integrity or dysfunctional host factors such as SAMHD1 and CPSF6 can result in cytoplasmic DNA accumulation, activating cGAS and inducing IFN and cytokine production. This mechanism also applies to endogenous retroviruses, where cGAS and STING are essential for effective immune responses. Furthermore, numerous intracellular bacteria, including *Mycobacteria, Legionella*, and *Listeria,* activate the cGAS-STING pathway primarily through the detection of bacterial DNA within the cytoplasm [30]. Although the precise mechanism by which bacterial DNA enters the cytoplasm remains unclear, the induction of interferons *via* cGAS-STING largely depends on cGAS, except for specific bacteria like *Listeria monocytogenes*. This emphasizes the fundamental role of this pathway in mediating immune responses against a wide range of pathogens.

Depression, one of the most prevalent mental disorders, has a complex pathogenesis. Medical research indicates that depression is not merely a mood or personality issue but a disease with a clear biological basis, resulting from a combination of genetic predispositions, abnormal neurodevelopment in early life, and adverse environmental factors later in life. Its pathogenesis involves alterations in the organization and structure of the central nervous system, the function of central neurotransmitters and their receptors, and neuroendocrine function. Numerous studies have confirmed that morphological and structural changes in the central nervous system (including the hippocampus, prefrontal cortex, and anterior cingulate cortex) are directly responsible for the development of depression [31, 32]. In recent years, the cGAS-STING pathway has emerged as a significant factor in central nervous system perceptual abnormalities, neuronal damage and repair, and neuroinflammatory regulation. For instance, impaired mitochondrial autophagy in various neurodegenerative diseases leads to the accumulation of mitochondrial DNA (mtDNA) in the cytoplasm, which in turn activates the cGAS-STING pathway. On one hand, excessive activation of the cGAS-STING pathway can cause neuronal cell damage or even death [33]. On the other hand, this signalling can drive age-related inflammation and neurodegeneration, ultimately affecting memory and other bodily functions [15]. Degrading neuronal cytosolic dsDNA or inhibiting cGAS-STING signal transduction can significantly reduce neuronal loss and improve neuroinflammation [34, 35].

## 3 THE ROLE OF cGAS-STING PATHWAY IN DEPRESSION

**Fig. (1):**
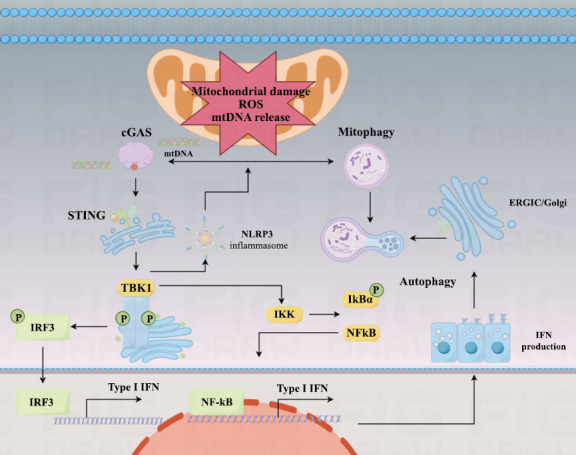
A schematic illustration depicting the signal transduction of cGAS-STING in depression. Upon binding to the mtDNA released from damaged mitochondria, cGAS undergoes conformational changes that facilitate the synthesis of cGAMP. This secondary messenger is subsequently recognized by STING, a protein located in the endoplasmic reticulum. Following its activation, STING translocates to the Golgi apparatus, where it forms a signaling complex by recruiting TBK1 and IRF3. TBK1 phosphorylates IRF3 and NF-κB activation. Consequently, the phosphorylated IRF3 migrates to the nucleus, initiating the transcription of IFN and ISGs. The activated NF-κB leads to the production of inflammatory cytokines (including TNF-α, IL-1β, and IL-6). The NLRP3 inflammasome, activated by elevated levels of ROS, is further upregulated by the STING protein. Furthermore, moderate STING activation also initiates autophagy to facilitate degradation processes.

Although some studies have indicated that inhibiting cGAS-STING signalling can alleviate symptoms of depression or mitigate chronic stress-induced depression-like behaviors, the precise regulatory mechanisms of cGAS-STING in depression remain to be fully elucidated. Given that cGAS-STING signalling is involved in various pathological pathways, its role in depression is likely to be complex (Fig. **1**). Therefore, this discussion will explore the function of cGAS-STING signalling in regulating neuroinflammation, oxidative stress, and autophagy within the context of depression and related neurological disorders.

### 3.1 cGAS-STING Signalling and Neuroinflammation in Depression

A substantial body of research has highlighted the strong association between cGAS-STING signalling and inflammation. Depression can be conceptualized as a neuroimmune disorder, with the primary physiological mechanism underlying its development linked to the activation of the inflammatory system. Inflammation serves as a defensive response of the body to various stimuli. The body activates peripheral immunity through the release of pro-inflammatory cytokines, leading to neuroendocrine immune dysfunction and the establishment of an inflammatory response model for depression. Levels of pro-inflammatory cytokines are positively correlated with the risk of developing depression and the severity of symptoms. Studies have shown that concentrations of TNF-α, IL-6, IL-1β, and CRP are elevated in patients with depression compared to healthy controls [36]. However, antidepressant medications have been found to reduce levels of TNF-α, IL-1β, IL-6, and CRP. The occurrence of mental disorders, including depression and anxiety, is associated with increased CRP levels. Linear regression analysis indicates that higher CRP levels correlate with greater overall severity of depressive symptoms [37]. Similarly, IL-6 levels are positively correlated with the severity of depressive symptoms [38]. Research has confirmed that IL-6 is a pro-inflammatory cytokine produced by the immune system in response to chronic inflammation. Among pro-inflammatory cytokines, TNF-α mediates the release of various cytokines, such as IL-6, IL-8, and IL-1β, by stimulating macrophages [39]. Prolonged exposure to TNF-α to enhance immune function has been associated with the development of severe psychiatric disorders, including major depression, as well as an increased HPA response and risk factors for immunotherapy-induced depression. In animal models, chronic stress has been shown to induce depression-like behaviors accompanied by elevated levels of pro-inflammatory cytokines. The administration of lipopolysaccharide (LPS), which activates the immune system and triggers inflammatory responses, can induce depressive-like behaviors in rodents [40]. Additionally, pro-inflammatory cytokines can decrease serotonin levels in the brain by activating indoleamine 2,3-dioxygenase, an enzyme responsible for metabolizing tryptophan [41].

Currently, clinically applied antidepressants can be categorized into selective serotonin and norepinephrine reuptake inhibitors (SNRIs), selective serotonin reuptake inhibitors (SSRIs), tricyclic antidepressants (TCAs), and monoamine oxidase inhibitors (MAOIs). There is a notable connection between these traditional antidepressant mechanisms and neuroinflammation. Venlafaxine, an SNRI, affects the central nervous system's inflammatory response, which is a significant mechanism underlying its antidepressant effects. Zhang *et al.* demonstrated that venlafaxine may reduce neuroinflammation by inhibiting microglial and astrocyte activation, thereby exerting antidepressant efficacy. SSRIs, such as citalopram, primarily act on the serotonin transporter to inhibit the reuptake of serotonin, increasing its concentration in the synaptic cleft and enhancing serotonin function. However, SSRIs have also been found to possess moderate immunomodulatory effects, improving mood in depressed patients by modulating immune function [42].

Additionally, minocycline, a second-generation tetracycline anti-inflammatory drug, has demonstrated strong antidepressant effects [43]. Furthermore, the potential antidepressant benefits of anti-inflammatory drugs have been widely investigated. A meta-analysis involving 30 randomized controlled trials with a total of 1,610 participants indicates that anti-inflammatory medications, whether used as adjunctive therapies or as monotherapy, exhibit significant antidepressant effects compared to placebo in individuals with MDD [44].

The specific pathways through which inflammation influences depression remain unclear. Evidence from various studies strongly suggests that microglia play key roles in the neuroinflammation associated with depression. Elevated microglial activation has been observed in both individuals with MDD and in animal models exhibiting depression-like behaviors [45]. These activated microglia are primary sources of inflammatory cytokines. It is hypothesized that the production of pro-inflammatory cytokines is predominantly regulated by the NF-κB signalling pathway, which is integral to the regulation of immune and inflammatory responses. Under stress conditions, IκBα undergoes phosphorylation at serine residues, leading to its ubiquitination and subsequent degradation. This degradation allows NF-κB to be phosphorylated by the activated IκB, facilitating its translocation into the nucleus. Within the nucleus, NF-κB promotes the expression of target genes, including IL-6, IL-1β, and TNF-α [46]. These pro-inflammatory cytokines, in turn, activate NF-κB, thereby amplifying the inflammatory response. Antidepressant medications, such as SSRIs and SNRIs, have been shown to reduce neuroinflammation by modulating the NF-κB inflammatory pathway and decreasing cytokine levels in blood and tissues.

In recent years, the relationship between the NLRP family, particularly the NLRP3 inflammasome, and depression has generated increasing attention. The NLRP3 inflammasome consists of NLRP3 and pro-caspase-1. The formation of this complex converts inactive pro-caspase-1 into active caspase-1, promoting the maturation and secretion of IL-1β and IL-18, leading to inflammation [47]. Research since 2014 suggests that factors such as chronic stress can activate NLRP3 inflammasomes in the brain, generating an inflammatory response that may be a key mechanism underlying depression-like behaviors. Consequently, NLRP3 has emerged as a new target for studying the mechanisms of inflammation and depression in the central nervous system. IL-1β induced by NLRP3 inflammasomes is essential for promoting learning, memory, and emotional responses [48]. However, excessively high levels of IL-1β can be excitotoxic, leading to abnormalities in synaptic structure and function, which are closely associated with the development of depression [49]. One study found that NLRP3 inflammasomes are involved in depression-like behaviors caused by chronic stress by upregulating IL-1β production in hippocampal tissue. The use of NLRP3 inflammasome inhibitors, such as amitriptyline, was effective in improving depressive symptoms in mice [50].

Additionally, chronic stress has been shown to induce activation of hippocampal NLRP3 inflammasomes and increase levels of IL-1β and IL-18, which in turn lead to depression-like behaviors in mice. These effects can be reversed by the specific inflammasome inhibitor VX-765 [51]. Furthermore, the activation of NLRP3 inflammasomes can regulate the synthesis of pro-inflammatory cytokines through signalling pathways such as MAPK, which subsequently affects central neuroinflammation and depression-like behaviors. These findings suggest that the NLRP3 inflammasome and its downstream signalling pathways play significant roles in neuroinflammation and depression.

Studies have shown that crosstalk exists between cGAS-STING, NF-κB, and NLRP3. The most recognized function of cGAS-STING is to mediate interferon activity. However, another primary signalling pathway engaged by STING involves transcriptional activation mediated by NF-κB. Activated STING recruits TBK1 to the STING complex through interaction with TBK1. In this complex, TBK1 is activated by phosphorylation, which in turn phosphorylates the transcription factor IRF3 and initiates interferon-I expression. It is well-known that TBK1 also activates NF-κB signalling. In addition to activating TBK1, activated STING also interacts with TRAF6 to activate the IκB kinase complex. The activated IKK complex catalyzes the phosphorylation of IκBα on specific serine residues (*e.g*., Ser32 and Ser36), tagging it for ubiquitination and proteasome-mediated degradation.

Degradation of IκBα deregulates the inhibition of NF-κB, allowing it to be released from the cytosol and translocated to the nucleus. STING-dependent NF-κB activation is not entirely contingent on the C-terminal tail (CTT). Although TBK1 can enhance NF-κB activation, it is not essential and can be substituted by IKKε, which operates upstream of TAK1 and IKK complexes in specific cell types. This is supported by the observation that ancestral STING homologues in insects and early metazoans lack the CTT signalling domain yet still facilitate host defense by activating NF-κB responses. This suggests that STING-dependent antimicrobial mechanisms have an evolutionary basis.

Additionally, certain vertebrate fish, such as zebrafish, have developed extra motifs appended to the CTT, which further enhance NF-κB activation by recruiting TRAF6 [52]. While STING-mediated transcription of NF-κB is a highly conserved and significant downstream function, the specific molecular interactions between STING and components of the NF-κB pathway remain unclear. Activation of the cGAS–STING pathway also triggers non-canonical NF-κB responses by inducing the nuclear translocation of p52–RELB [53]. This signalling pathway, which inhibits type I interferons and canonical NF-κB activity, acts as a crucial negative regulator of STING effector functions, with important implications for cancer immune evasion and metastasis. Moreover, under certain conditions, cGAS–STING signalling may affect p53, MAPK, and STAT3 pathways, suggesting additional mechanisms by which STING can influence cytokine production. Although STING-dependent NF-κB activation involves multiple signalling modules beyond the CTT, the precise molecular mechanisms remain partially understood. The evolutionary conservation of this pathway underscores its importance in immune defence, while its interaction with various signalling proteins highlights the complexity of its regulation. Further research is necessary to elucidate the detailed interactions and regulatory mechanisms, which could provide deeper insights into immune responses and potential therapeutic targets for diseases involving dysregulated NF-κB activity.

NLRs significantly influence the initiation of inflammatory responses. Research has established that various members of the NLR family, including NLRP1b, NLRP3, NLRC4, NLRP6, and NLRP12, participate in the assembly of inflammasomes and play a regulatory role in innate immunity. Upon stimulation by cytosolic DNA, STING interacts with NLRP3, promoting inflammasome activation through two distinct mechanisms. On the one hand, STING recruits NLRP3 and facilitates its localization to the endoplasmic reticulum, enhancing the assembly of the inflammasome complex [54]. On the other hand, STING engages with NLRP3 to reduce its polyubiquitination at lysine residues K48 and K63, thereby activating the inflammasome. It is well-established that the assembly of the NLRP3 inflammasome leads to the activation of caspase-1, which subsequently triggers the production of various pro-inflammatory cytokines. Caspase-1 acts as a critical mediator linking the inflammasome to the generation of inflammatory cytokines. The activation of NLRP3 by the cGAS-STING pathway involves the recruitment and post-translational modification of NLRP3, leading to inflammasome assembly and subsequent cytokine production. The interaction between caspases and cGAS highlights a complex regulatory network that governs the immune response to cytoplasmic DNA, suggesting potential targets for therapeutic intervention in inflammation.

Recently, a study found that cGAS-STING signalling is involved in astrocyte inflammatory injury in mouse models of depression [55]. UNC9995 promotes the binding of β-arrestin2 to NLRP3, thereby inhibiting the activation of the NLRP3 inflammasome and subsequently preventing neuronal degeneration [56]. Inflammatory signalling, including the cGAS–STING pathway, is activated in chronic social defeat stress-induced depressive mice. UNC9995 functions as a Drd2/β-arrestin2-biased agonist, inhibiting cGAS/STING activation through interaction with the JAK/STAT3 signalling pathway, thereby mitigating depressive behaviors in the chronic social defeat stress model. Recent studies indicate a strong association between cGAS-STING-NLRP3 signalling and symptoms of depression [16]. Treatments such as electroacupuncture (EA) and cGAS-shRNA markedly reduced depressive behaviors in chronic unpredictable mild stress (CUMS) mice, accompanied by improved hippocampal pathology, decreased levels of TNF-α, IL-1β, and IL-6, elevated concentrations of serotonin (5-HT) and norepinephrine (NE), and inhibited microglial activity.

Additionally, the CUMS+EA and CUMS+cGAS-shRNA groups exhibited significantly higher expression levels of Bax, cGAS, STING, TBK1, IRF3, and NLRP3, along with reduced expression of Bcl-2. EA markedly reduced depressive behaviors in mice, a consequence strongly related to decreased neuroinflammation, elevated monoamine levels, suppressed microglial activation, and the downregulation of the cGAS-STING-NLRP3 signalling pathway. Another study reported that silibinin could inhibit cGAS-STING-induced neuroinflammation and hippocampal damage, thereby ameliorating depression-like behaviors in animal models of Parkinson's disease [19]. Thus, there appears to be a positive feedback loop between cGAS-STING signalling and inflammatory cytokines in depression, and the underlying mechanism is worth an in-depth study.

Ameliorating depression through non-pharmacological interventions is of great importance. Many studies have found exercise to be one of the most effective treatments for depression. Molecular mechanisms of improving depression through exercise involve neurotransmitter regulation, nerve growth factor modulation, inflammatory suppression, stress response modulation, and structural alternation in the brain [57-60]. Strikingly, several recent studies have shown that exercise is involved in the regulation of cGAS-STING activity, thus further affecting inflammation and tissue damage [20, 21]. Following a high-fat diet (HFD), the expression levels of cardiac STING and NLRP3 are elevated, whereas aerobic exercise leads to a reduction in their expression. Activation of STING diminishes the protective benefits of aerobic exercise against HFD-induced cardiac dysfunction, pyroptosis, and inflammation. This study demonstrates for the first time that aerobic exercise alleviates cardiac dysfunction, pyroptosis, and inflammation through the inhibition of the STING-NLRP3 signalling pathway. Moreover, a single bout of moderate-intensity exercise suppresses mtDNA-induced STING activation and inhibits inflammatory responses by decreasing both hepatic cytoplasmic and circulating mtDNA levels.

In contrast, repeated bouts of exhaustive exercise enhance innate immune signalling through an increase in circulating mtDNA levels. However, the exact mechanism of infection remains to be elucidated. In the future, a comprehensive study of the upstream molecular mechanisms of STING signalling in animal models of depression modulated by aerobic exercise is necessary.

### 3.2 cGAS-STING Signalling and Oxidative Stress in Depression

Redox homeostatic imbalance is observed in mental disorders, and normal redox balance is essential for maintaining central nervous system (CNS) function. When redox imbalance occurs, particularly during oxidative stress, lipids, proteins, and DNA within functional cells can be damaged, potentially leading to cell death [61]. Neurons, astrocytes, and microglia in the brain contain a high number of mitochondria and NADPH oxidase (NOX) enzymes, enabling them to produce significant amounts of ROS. The brain is a highly oxygen-consuming organ with a large cortical surface area, rich in unsaturated fatty acids but deficient in antioxidant enzymes, resulting in low antioxidant capacity and increased susceptibility to oxidative stress [62]. Meta-analysis results indicate that oxidative stress levels are elevated while antioxidant defenses are impaired in patients with depression [63].

Furthermore, levels of oxidative stress markers such as F2-isoprostanes and 8-hydroxy-2'-deoxyguanosine (8-OHdG) are significantly elevated in individuals with depression [64]. Animal studies have identified stress-induced abnormalities in specific brain regions, such as the prefrontal cortex (PFC) and hippocampus, in depressed rats. These abnormalities include decreased glutathione (GSH) and superoxide dismutase (SOD) activity, along with elevated levels of ROS, malondialdehyde (MDA), and carbonyl compounds resulting from chronic unpredictable mild stress (CUMS), chronic restraint stress, and chronic social isolation [65]. Moreover, studies have confirmed the antioxidant properties of antidepressants and the antidepressant effects of antioxidants in both depressed individuals and animal models. For instance, the antidepressants fluoxetine and citalopram have been shown to increase SOD activity and decrease MDA and carbonyl levels in patients with MDD and in rats subjected to chronic restraint stress. Similarly, the antioxidant diallyl disulfide has been found to reverse lipopolysaccharide (LPS)-induced depressive-like behaviors in mice [66]. These findings underscore oxidative stress as a significant factor in the pathogenesis of depression and suggest that enhancing antioxidant activity could represent a promising therapeutic strategy.

The underlying mechanisms of oxidative stress-induced depression are complex. A comprehensive analysis of numerous studies suggests that potential factors include mitochondrial dysfunction and associated ATP depletion, central glutamate excitotoxicity, disruptions in brain-derived neurotrophic factor (BDNF)/tyrosine receptor kinase B signalling, serotonin deficiency, disturbances in the microbiota-gut-brain axis, and dysregulation of the hypothalamic-pituitary-adrenal (HPA) axis [67]. Commonalities among these mechanisms include imbalances in intracellular ion homeostasis, cytokine dysregulation, impaired intracellular material transport, and functional cell death. Since ROS are primarily produced by mitochondria and accumulated ROS can damage mitochondria, it is crucial to focus on mitochondria-related functions. A growing body of evidence suggests that mitochondrial dysfunction is present in depression [1]. Various mitochondrial abnormalities have been identified in both individuals with depression and animal models of the condition. For instance, ATP production levels are diminished in the brains and peripheral blood mononuclear cells of depressed patients compared to the controls [68].

Additionally, increased deletions and mutations of mtDNA, along with a reduced mtDNA copy number, have been detected in individuals with depression [69]. In mice subjected to chronic mild stress-induced depression, decreased activity of mitochondrial enzymes, compromised mitochondrial membrane potential, and altered mitochondrial ultrastructure have been observed [70]. These alterations may lead to reduced mitochondrial biogenesis, increased membrane permeability, and elevated mitochondrial ROS production, ultimately resulting in impaired neurogenesis, decreased synaptic plasticity, and increased apoptosis.

In the classical cGAS-STING signalling pathway, the downstream signal ISG15 participates in regulating lipid peroxidation and ROS accumulation [13]. ISG15, a member of the interferon-stimulated gene (ISG) family, plays a role in inducing interferon expression, facilitating “protein ISGylation,” and interfering with ubiquitin modifications. Through ISG15, STING can negatively influence the ubiquitin-proteasome system, leading to an increase in interferon-mediated ROS [71]. Indeed, interferon-driven protein ISGylation modulates the ubiquitin-proteasome system to elevate cellular ROS levels. Additionally, ISG15 inhibits glutathione peroxidase, an antioxidant molecule that mitigates oxidative stress. Suppression of STING enhances glutathione peroxidase (GPX) activity by inhibiting ISG15. Recently, Hayman *et al.* reported that reducing STING expression led to decreased levels of ISG15 and ROS-associated genes, such as HECT domain and RCC1-like domain-containing protein 5 (HERC5), Kruppel-like factor 4 (KLF4), and dual oxidase 2 (DUOX2) [72]. These findings indicate that STING acts as an upstream regulator of intracellular oxidative processes. Another study showed that glutathione peroxidase 4 is indispensable for cGAS-STING activation [73]. The inactivation of GPX4 did not influence the binding of DNA to cGAS; however, it inhibited the transport of STING to the Golgi apparatus by promoting the carbonylation of STING at C88. An interesting study demonstrated that moderate-intensity treadmill exercise increases GPX4 levels and decreases the accumulation of lipid peroxides partly *via* suppressing the STING pathway after traumatic brain injury [74]. In addition, swimming training elevates the levels of miR-17-3p, which in turn increases the GPX4 level and activates the KEAP1/NRF2 pathway while inhibiting the cGAS/STING pathway [21]. Actually, Nrf2 is also a promising target for treating depression [1]. Some vitagenes (including heat shock proteins, HSPs; heme oxygenase 1, HO-1; and sirtuin, SIRT, family) closely link oxidative and nitrosative stress and Nrf2-associated resilience network within the central nervous system [75]. It is widely known that SIRT1 can activate antioxidant transcription factors through deacetylation and enhance cellular antioxidant defense. HO-1 is one of the core enzymes in the regulation of oxidative stress and plays a key role in the maintenance of REDOX balance by degrading heme to produce metabolites with antioxidant, anti-inflammatory, and cell protective effects. Nrf2 promotes mitochondrial biogenesis and maintains mitochondrial homeostasis by regulating SIRT1 and HO-1 expressions [76]. Other indirect evidence suggests that nitrogen-oxygen stress induces HO-1 and HSPs in brain cells and that the addition of specific compounds to regulate Nrf2 activity can reverse their expression levels [77, 78]. Understanding the crosstalk between the cGAS-STING pathway and Nrf2 signalling helps to offer new insights for the treatment of depression. Oxidative stress is consistently linked to mitochondrial dysfunction, leading to damage and leakage of mtDNA, which subsequently activates the cGAS-STING pathway. A recent study demonstrated that chronic ethanol exposure leads to microglial activation, significantly elevated cytoplasmic mitochondrial DNA levels, and mitochondrial dysfunction in the PFC of mice [79]. Treatment with the STING inhibitor H-151 helps suppress microglial activation and improve mitochondrial dysfunction. Another study found that betaine, a natural neuromodulator, can alleviate depression-like behaviors in dextran sulphate sodium-treated mice and repair neurogenesis in the hippocampus by suppressing DNA damage and mitochondrial dysfunction to block the cGAS-STING signalling pathway [80]. These findings suggest that cGAS-STING signalling activity and mitochondrial dysfunction are causally related to the symptoms of depression or depression-like behaviors. The causal link between oxidative stress and the activation of the cGAS-STING signalling pathway in the onset and progression of depression remains unresolved.

Additionally, there is ongoing debate regarding whether oxidative stress promotes STING activity or inhibits its activation. Moreover, few studies have examined the direct interactions between the cGAS-STING pathway and oxidative intermediates. Consequently, further investigation is required to clarify their relationship. Nonetheless, it is acknowledged that changes in oxidative stress levels impact cGAS-STING signalling and the underlying pathology of depression.

### 3.3 cGAS-STING Signalling and Autophagy in Depression

Autophagy, a critical intracellular metabolic process, protects cells and organisms by degrading and recycling surplus or potentially harmful cytosolic components, thereby preventing the accumulation of toxic proteins and damaged organelles. Under physiological conditions, autophagy is beneficial and essential for maintaining cellular homeostasis. Dysfunction of autophagy is closely associated with the development of tumors, neurodegenerative disorders, metabolic diseases, and immune-related conditions. Moreover, autophagy contributes to the growth and maturation of neuronal axons, dendrites, and synapses in both developing and mature brains. In recent years, an increasing number of studies have suggested a close relationship between autophagy dysregulation and depression. Several studies indicate that changes in gene expression within the PFC of individuals with MDD correlate with the activation of microglia and the regulation of autophagy [81]. Notably, MDD patients demonstrate elevated levels of autophagy-related genes, including LC3, ATG12, and Beclin 1, in their peripheral blood mononuclear cells [12].

Additionally, gene set enrichment analysis of plasma samples has implied an association between autophagy and postpartum depression [82]. MTOR serves as a vital energy sensor and acts as an inhibitor of the autophagy process. The mTOR signalling pathway has been identified in the postmortem brains of individuals diagnosed with MDD [83]. The AMPK/mTOR, PI3K/AKT/mTOR, and MAPK/mTOR pathways have all been implicated in the course of depression [84-86]. Regulating mTOR activity to control the degree of autophagy is currently a popular target for antidepressant drug development. Furthermore, other autophagy receptors or markers, such as p62, Beclin-1, and LC3B, are also considered potential targets for antidepressant drugs [87, 88]. Consequently, the therapeutic effects of both traditional and novel antidepressants are linked to the regulation of autophagy, suggesting that targeting autophagy could be a promising strategy for enhancing depression treatment.

Earlier studies primarily focused on the mechanisms of IFN production and inflammatory induction by the cGAS-STING pathway. However, as research progressed, it became evident that autophagy induction is an evolutionarily conserved function of cGAS-STING signalling. Saitoh *et al.* were the first to demonstrate that dsDNA can trigger the co-localization of STING, ATG9a, and LC3, all of which are essential proteins involved in the autophagy process [89]. Following this discovery, STING was identified as a key factor that initiates autophagy in response to microbial DNA, facilitating the degradation of pathogens by directing them to autophagosomes. Additionally, research has shown that cGAS can directly bind to the coiled-coil domain of Beclin-1, a critical protein for the initiation of autophagy [90]. This binding interaction inhibits the production of cGAMP and IFN while promoting the degradation of cytosolic DNA through autophagy by releasing Rubicon from the Beclin-1 complex. Importantly, Gui and colleagues elucidated a mechanism by which STING mediates autophagy without activating TBK1 or inducing IFN [25]. In this pathway, cGAS detects cytosolic DNA and synthesizes cGAMP, which subsequently binds to STING. This binding causes STING to translocate to the endoplasmic reticulum-Golgi intermediate compartment (ERGIC) through its interaction with SEC24C. The ERGIC then serves as a membrane source for LC3 lipidation, promoting the formation of autophagosomes. In various invertebrates, such as *Drosophila* and the sea anemone *Nematostella vectensis*, STING is involved solely in the induction of autophagy and does not participate in the IFN response [91]. These studies suggest that the induction of autophagy is an evolutionarily conserved function of the cGAS-STING signalling axis, existing prior to the emergence of IFN signalling.

Furthermore, structural analyses have revealed that STING contains a conserved LC3-interacting region (LIR) domain, which becomes exposed to the cytoplasm following conformational changes upon activation [92]. As a result, the exposed LIR domain can directly interact with LC3 to activate autophagy, leading to the degradation of STING itself and phosphorylated TBK1 (p-TBK1). This finding also demonstrates that STING can directly link immune activation to autophagy. Additional studies have shown that STING orchestrates endoplasmic reticulum stress and the unfolded protein response through a novel UPR motif within its cyclic-dinucleotide-binding (CBD) domain. This motif exerts a negative regulatory effect on the Akt/tuberous sclerosis complex (TSC)/mTOR pathway, thereby enhancing canonical autophagy [93].

Since cGAS-STING signalling is closely linked to endoplasmic reticulum stress, autophagy, and neuroinflammation, research is increasingly focusing on the function of the cGAS-STING-autophagy axis in depression [94]. Recently, a study found that activation of the cGAS-STING signalling pathway under manganese stimulation could affect the clearance of toxic proteins *via* the autophagy-lysosomal pathway in microglia and the brain [95]. It has been confirmed that exposure to environmental air manganese levels of 203 ng/m^3^ or higher is linked to cognitive impairments, depression, and anxiety [96]. Manganese exposure activated the cGAS-STING pathway and triggered dysfunction of the autophagy-lysosomal pathway. Blocking the cGAS-STING pathway restores the function of the autophagy-lysosomal pathway and clears harmful proteins in microglia. Moreover, inhibiting the cGAS-STING pathway can restore the function of the autophagy-lysosomal pathway and offer protection to the CNS. Mitophagy, a form of selective autophagy, is associated with the pathophysiology of depression [10]. Mitochondrial dysfunction has been recognized as a significant contributor to depression [97]. Mitophagy is a key pathway for removing damaged mitochondria, thereby ameliorating mitochondrial dysfunction. Following the administration of antidepressant medication in a mouse model of depression, mitophagy was found to be induced [98]. Targeting mitophagy may represent an effective strategy for treating depression [99].

On the other hand, mitophagy inhibits mtDNA release, thus suppressing the hyperactivation of cGAS-STING signalling in the brain [33]. Therefore, we hypothesize that mitophagy may downregulate neuroinflammation and CNS injury by inhibiting cGAS-STING signalling activity in the onset and progression of depression. Although direct evidence that cGAS-STING signalling regulates depression through interactions with autophagy remains relatively lacking, it is worthy of being penetratingly explored.

### 3.4 The Crosstalk between cGAS-STING Signalling and Inflammation-oxidative Stress-autophagy Axis in Depression

Studies have demonstrated complex interactions among inflammation, oxidative stress, and autophagy in the onset and progression of depression. A substantial body of research suggests that targeting autophagy to combat neuroinflammation may be an effective strategy for treating depression [100]. For instance, deletion of the autophagy regulator ATG7, specifically in microglia, leads to heightened accumulation of intracellular phagocytic myelin and the progression of multiple sclerosis, indicating that the capacity of microglia to eliminate tissue debris is crucial for reducing inflammation in the CNS [101]. The regulatory role of autophagy-related genes (ATGs) in inflammation suggests that appropriate autophagy can attenuate neuroinflammation. Moreover, autophagy is essential for preventing inflammation-related neuronal damage and cell death. Extracellular vesicles derived from neural stem cells can effectively suppress microglial activation and neuroinflammation by stimulating autophagy, ultimately leading to decreased spinal cord injury, enhanced neuronal recovery, and reduced cell death [102]. Deletion of microglial ATG5 can result in deficits in motor coordination and cognitive learning, as well as a decrease in tyrosine hydroxylase neurons, accompanied by increased neuroinflammation and lowered dopamine levels in the striatum induced by the activation of the NLRP3 inflammasome and phosphodiesterase 10A-cyclic adenosine monophosphate [103]. In fact, moderate autophagy helps inhibit NLRP3 activation, suppress IL-1β production, and further improve MDD [104]. In turn, inflammatory signalling can also influence the autophagy process through various pathways.

Among these, TNF-α and IL-1β can suppress autophagy by activating the NF-κB signalling pathway [105]. The function of cGAS-STING as a bridge connecting autophagy and inflammation during infection has been demonstrated [13]. The cGAS-STING signaling is often in a state of hyperactivation in the brains of animal models of depression [16, 18]. Inhibition of the cGAS-STING pathway can restore autophagy-lysosomal function and alleviate neuroinflammation. Whether this effect is parallel or reciprocal warrants further investigation. Chronic inflammation, excessive production of inflammatory mediators, and dysregulated inflammatory signalling are key characteristics of the inflammatory response observed in individuals with MDD. Autophagy is crucial for modulating these inflammatory responses. Gaining insight into these mechanisms not only helps elucidate the pathological processes underlying depression but also offers potential new targets for therapeutic intervention in mental disorders.

Autophagy is vital for maintaining redox homeostasis. Both macroautophagy and selective autophagy contribute to reducing oxidative stress by degrading damaged mitochondria and harmful proteins, thereby decreasing the production of ROS in the brain [11]. Likewise, oxidative stress can influence the autophagy process through various pathways. ROS can enhance autophagy by activating AMPK and suppressing mTOR. However, excessive oxidative stress may inhibit autophagy by damaging proteins associated with the autophagy process [106]. The mTOR signalling pathway is crucial in mediating the relationship between autophagy and redox homeostasis. Research indicates that activation of the mTOR pathway can exacerbate oxidative stress by suppressing autophagy. Conversely, inhibiting the mTOR pathway can alleviate oxidative stress by enhancing autophagic activity [11]. Many studies have reported decreased mTOR activity in the brains of animal models of depression or in patients with depression [107]. Mice and rats subjected to chronic unpredictable stress (CUS) display depressive-like behaviors, which correlate with decreased phosphorylation levels of mTOR and its downstream signalling molecules, including phosphorylated p70S6K, in various regions such as the prefrontal cortex, hippocampus, and amygdala [108].

Additionally, the knockout of mTOR in mice recapitulates the depressive-like behaviors triggered by CUS [109]. Research conducted by Jernigan examined the expression levels of mTOR and its downstream signalling targets in the prefrontal cortex of individuals with depression and healthy controls. The findings revealed decreased protein expression of mTOR, p70S6K, eIF4B, and phosphorylated eIF4B in subjects with MDD compared to the control group [83]. This suggests a deficiency in mTOR-dependent signalling, which may impair the downstream targets responsible for the translation of synaptic proteins. Many drugs targeting mTOR show favorable antidepressant effects, such as ketamine. It is widely believed that mTOR is one of the most critical signals regulating autophagy. It has been confirmed that suppressing excessive autophagy by activating mTOR signalling could alleviate chronic unpredictable mild stress-induced depressive-like behavior [87]. A study showed that resveratrol could simultaneously reverse increased lipid peroxidation and decreased phosphorylation of Akt and mTOR in rat models of depression, further exerting antidepressant properties [110]. However, this study did not establish a link between autophagy and oxidative stress in this process.

Additionally, mitochondrial dysfunction has been identified in multiple brain regions of individuals with MDD.The accumulation of damaged mitochondria accelerates neuronal dysfunction. These impaired mitochondria exacerbate changes in the brain's microenvironment, leading to increased neuroinflammation and energy depletion, which in turn aggravates the development of depression [111]. Mitophagy serves as an initial protective mechanism for nerve cells, facilitating adaptation to cellular stress by removing damaged and aging mitochondria. In a mouse model of depression, fluoxetine attenuated cell death by enhancing the clearance of impaired mitochondria, promoting mitophagic flux in astrocytes, and decreasing the accumulation of mtROS [98]. Moreover, insufficient mitophagy activity results in the failure of mitochondrial quality control mechanisms, leading to the subsequent activation of the caspase cascade during the later phases of cold restraint stress. This ultimately causes NF-κB-dependent pro-inflammatory mucosal damage and Bax-dependent apoptosis. Studies have shown that G11-5 and fluoxetine can alleviate depressive behaviors in mice, as evidenced by a reduction in escape failures in the learned helplessness model and higher social interaction in the social defeat stress model. This improvement is attributed to their protective effects on mitophagy, as well as the restoration of retrograde axonal transport and neurotransmitter release [112, 113]. This selective autophagy modulates neuroinflammation and intracellular redox homeostasis during the development of depression. Mitochondrial dysfunction leads to the release of mtDNA from glial cells into the cytoplasm, where it is detected by the cGAS-STING signalling pathway, resulting in the upregulation of inflammatory factors.

**Fig. (2):**
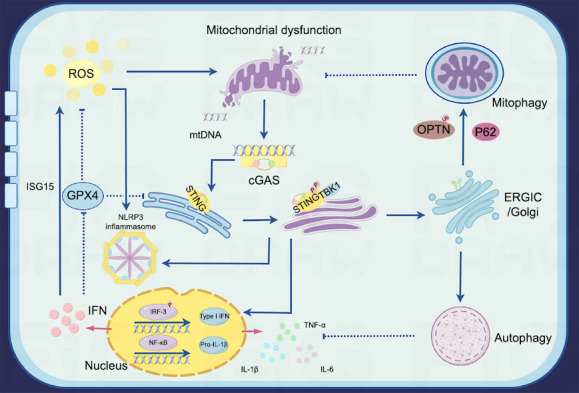
Schematic representation of the interaction between the cGAS-STING pathway and inflammation, oxidative stress, and autophagy in depression. Mitochondrial DNA can be recognized by the cGAS-STING signaling pathway, leading to the induction of pro-inflammatory cytokines (including TNF-α, IL-1β, and IL-6) and IFN production through the TBK1-IRF3/NF-κB/NLRP3 pathway. Moreover, the activated STING translocates to ERGIC through its interaction with SEC24C. The ERGIC then serves as a membrane source for LC3 lipidation, promoting the formation of autophagosomes. TBK1 can also lead to the ubiquitination of OPTN, which is another key autophagy-related protein. Additionally, mitochondrial dysfunction triggers lipid peroxidation and oxidative stress, which can inactivate STING by promoting carbonylation at C88. Notably, GPX4 serves as a critical link between the cGAS-STING axis and oxidative stress. Specifically, the activation of GPX4 mitigates oxidative stress, ensuring that activated STING is successfully translocated to the Golgi apparatus for subsequent action. Conversely, the activation of the cGAS-STING-IFN axis enhances oxidative stress and inhibits GPX4 activity through the expression of ISG15. Furthermore, ROS inhibits STING dimerization by oxidizing cysteine 147 on the STING protein. Thus, redox modification of STING represents a significant regulatory mechanism influencing STING activity. Mitophagy, on the other hand, reduces intracellular mtDNA levels by removing damaged mitochondria, ultimately inhibiting cGAS-STING signaling activity. Given its ability to regulate IFN, inflammation, oxidative stress, and autophagy, the cGAS-STING pathway presents a promising target for alleviating depressive symptoms.

Additionally, mtDNA-triggered activation of the cGAS-STING pathway can exacerbate neuronal death associated with PANoptosis by modulating endoplasmic reticulum stress [114]. These factors are key contributors to mental disorders (Fig. **2**). Some reports have indicated the antidepressant potential of autophagy and cGAS-STING signalling modulators, but the exact mechanisms remain unclear [18, 115, 116]. More studies are needed to analyze the positive and negative feedback loops between cGAS-STING signalling and inflammation, oxidative stress, and autophagy.

## CONCLUSIONS AND PROSPECTS

There is substantial evidence suggesting that neuroinflammation, oxidative stress, and autophagy dysregulation resulting from mitochondrial dysfunction significantly contribute to the development of depression. Alleviating mitochondrial dysfunction has beneficial effects on the treatment of depression. The mtDNA released from damaged mitochondria activates the cGAS-STING signalling pathway, which holds great promise for depression therapy. On the one hand, cGAS-STING activity contributes to the production of IFN and pro-inflammatory cytokines, thereby inducing neuroinflammation. On the other hand, inappropriate activation of cGAS-STING triggers dysfunction of the autophagy-lysosomal pathway and programmed cell death. Moreover, cGAS-STING signalling and oxidative stress are interconnected, influencing each other in a cause-and-effect manner. Therefore, cGAS-STING signalling plays a crucial role in regulating multiple pathways. Focusing on the cGAS-STING pathway has yielded promising outcomes in several models of depression.

Nevertheless, given that cGAS-STING signaling is involved in multiple biological processes, the efficacy of cGAS/STING activators and inhibitors for treating depression requires thorough investigation. Additionally, some non-pharmacological interventions, such as physical exercise, have been reported to regulate the cGAS-STING pathway. For instance, exercise leads to the downregulation of the mitochondrial disulfide relay carrier CHCHD4, resulting in decreased import of TP53-regulated inhibitor of apoptosis 1 into the mitochondria. This reduction can lower cardiolipin levels and promote the oligomerization of VDAC in skeletal muscle. The oligomerization of VDAC is associated with the release of mtDNA, which subsequently activates the cGAS-STING/NF-κB innate immune signalling pathway [20]. Moreover, a 6- to 8-week aerobic exercise regimen (such as high-intensity interval training or moderate-intensity continuous training) alleviates mental disorders, pyroptosis, and neuroinflammation by targeting the cGAS-STING signalling pathway [117, 118]. In addition, swimming training elevates the levels of miR-17-3p, which in turn increases the GPX4 level and activates the KEAP1/NRF2 pathway while inhibiting the cGAS/STING pathway [21]. These efforts to modulate cGAS-STING signalling through non-pharmacological interventions to ameliorate depression are also well worth pursuing in depth.

It is widely believed that cGAS primarily resides in the cytosol, where it detects double-stranded DNA. In this compartment, DNA mainly originates from compromised mitochondria and invading pathogens. Consequently, this study examines how cGAS-STING activation-whether triggered by infections or mitochondrial damage-relates to depression. However, a key aspect of the DNA-related phenomena in depression involves the function of nuclear DNA within neural cells, a topic that spans a complex network of biological mechanisms and various regulatory factors. Research indicates that oxidative stress can provoke persistent damage to telomeric DNA, which may result in chromosomal instability and alterations in nuclear structure. This telomere impairment could contribute to the cellular senescence and functional decline observed in individuals with depression [119]. Moreover, the accumulation of DNA lesions appears to diminish the pluripotency of mesenchymal stromal cells, potentially restricting their clinical utility and linking them to features of tissue aging [120]. Additional findings have uncovered extensive correlations between polygenic risk scores for depression and DNA methylation patterns in genes involved in immune responses and neurodevelopment [121]. Chromatin fragmentation resulting from DNA damage may also translocate into the cytoplasm during mitotic slippage, a process marked by incomplete cell division following failed mitotic checkpoint resolution. Since nuclear DNA damage in this manner leads to the entry of its DNA into the cytoplasm, we believe that its effect on cGAS-STING signalling is similar to the situation this study discussed previously. In recent years, there has been increasing evidence that cGAS is also located in the nucleus and is associated with different nuclear substructures such as nucleosomes, DNA replication forks, double-strand breaks, and mitoses [122]. In the nuclear environment, cGAS binds to chromatin through interactions with the H2A–H2B dimer in nucleosomes, effectively sequestering it and preventing access to adjacent DNA necessary for active dimer formation [123]. Moreover, phosphorylation at the N-terminal region and specifically at the S291 residue of its C-terminal domain further restricts cGAS activation during mitosis [124]. A recent study found that HSV-1 infection induced the release of cGAS from the chromatin into the nuclear soluble fraction, where it could continue to mediate innate immune response in a nuclear STING-dependent manner [125]. This study implies that there should be more attention to the intranuclear localization of cGAS in patients with depression.

On the other hand, increasing evidence suggests that cGAS participates in the DNA damage repair process [126]. Nuclear cGAS is recruited to the site of damage through direct interaction with PARP1, independent of its enzymatic activity. This interaction disrupts the binding between PARP1 and the DNA repair protein Timeless, consequently inhibiting PARP1-mediated homologous recombination repair [127]. Moreover, the dimerization of cGAS and its association with chromatin are crucial for its role in DNA repair. Specifically, cGAS dimerization facilitates chromatin condensation, which in turn influences the RAD51-mediated strand invasion step. This ultimately impacts D-loop formation during the homologous recombination repair process, leading to a reduction in HR efficiency [128]. It is widely known that patients with depression have more DNA breaks and oxidative DNA damage, which may be caused by impairments of the DNA repair systems [129, 130]. Therefore, a deeper understanding of the function of nuclear cGAS is likely to provide new insights for the treatment of depression from the perspective of DNA damage repair.

Despite significant advancements in neuroscience over recent decades, the pathophysiology of MDD remains incompletely understood. Extensive research has identified multiple mechanisms implicated in MDD, including alterations in serotonergic, noradrenergic, dopaminergic, and glutamatergic neurotransmitter systems, elevated inflammation, oxidative stress, mitochondrial dysfunction, HPA-axis dysregulation, autophagy dysregulation, and diminished neurogenesis and neuroplasticity [131]. However, these pathological features are not consistently present across all individuals with MDD, and therapeutic interventions targeting these mechanisms have been only partially explored. The phenotypically distinct biological basis of depression suggests that MDD may encompass several distinct biological subtypes. A growing body of research indicates that all of these pathways are interconnected. Integrating pattern recognition receptor activation, neuroinflammation, redox homeostasis, and programmed cell death in depression can provide several benefits. Primarily, a single approach that isolates specific biological pathways without acknowledging other interactions risks overlooking the inherent complexity of the disorder. Secondly, a comprehensive model could account for the significant heterogeneity observed among individuals with MDD, wherein only subsets exhibit particular pathological characteristics. Thirdly, this integrative framework would guide future research toward investigating the intricate interactions among these pathophysiological factors rather than examining them in isolation. By recognizing the interactions between different biological systems, a theory of depression that encompasses a broader range of pathogenic mechanisms will help to understand the disease more accurately. This holistic perspective is essential for addressing the multifaceted nature of MDD, potentially leading to more effective and personalized treatment strategies.

## STUDY LIMITATIONS

Although there have been numerous reports indicating that the cGAS-STING signalingpathway and the associated physiological processes are involved in neurodegenerative diseases, as well asneuroinflammation and programmed cell death of nerve cells, there are still few studies on the direct impact ofthe cGAS-STING signal on the course of depression. This highlights the forward-looking and enlightening natureof this review.

## LIST OF ABBREVIATIONS

ATGs: Autophagy-related Genes
BDNF: Brain-derived Neurotrophic Factor
CNS: Central Nervous System
CRP: C-reactive Protein
CUMS: Chronic Unpredictable Mild Stress
EA: Electroacupuncture
HPA: Hypothalamic-pituitary-adrenal
IL-6: Interleukin 6
IRF3: Interferon Regulatory Factor 3
ISG: Interferon-stimulated Gene
MAOIs: Monoamine Oxidase Inhibitors
MDD: Major Depressive Disorder
PFC: Prefrontal Cortex
PRR: Pattern Recognition Receptor
ROS: Reactive Oxygen Species
SOD: Superoxide Dismutase
SSRIs: Selective Serotonin Reuptake Inhibitors
TCAs: Tricyclic Antidepressants
TNF-α: Tumor Necrosis Factor α
